# Optimizing Infraorbital Hollows Treatment With Hyaluronic Acid Fillers: Overview of Anatomy, Injection Techniques, and Product Considerations

**DOI:** 10.1093/asjof/ojaf069

**Published:** 2025-06-20

**Authors:** Tahera Bhojani-Lynch, Philippe Berros, Philippe Snozzi

## Abstract

Given the importance of the periorbital area in facial expression and as an indicator of age, facial rejuvenation often needs to address infraorbital hollowing. Hyaluronic acid (HA) has become a treatment of choice in attenuating infraorbital grooves and smoothening the eyelid-cheek junction. This article (1) presents the definition and origin of infraorbital age-related landmarks, namely the midface, tear trough and palpebromalar groove, (2) reviews the anatomical characteristics and the course of neurovascular structures to delineate the treatment area, and (3) emphasizes the importance of choosing the right injection techniques and products for safe, effective, and natural-looking rejuvenation of the infraorbital area. Patient selection should rely on defined criteria, weighing the procedure's benefit vs the risk of implant irregularities or visibilities. To achieve natural-looking results, it is recommended to first address volume loss in the midface. If the outcomes are not satisfactory, direct treatment of infraorbital hollow can be performed using a maximum filler volume of 0.5 mL per eye, administered at the supraperiosteal level, through either a serial puncture injection or linear threading technique with a needle, or by using retrograde or anterograde linear threading or deposition of microboluses with a cannula. The choice of the HA filler should be based on its ability to adapt during facial movement; the filler should therefore be smooth, malleable, and present limited hygroscopicity.

**Level of Evidence: 5 (Therapeutic)**: 
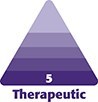

The eyes and periorbital region are central to many facial rejuvenation strategies. This is explained by their essential role in conveying emotions through facial expressions and giving an overall indication of the aging status and the sense of fatigue of the entire face.^1,2^ Requests for infraorbital treatments are usually focused on the correction of dark circles, tear troughs (TTs), or eye bags, which result in the perception of tiredness/sadness, and contribute to an aged appearance.^1^

Facial aging involves a dynamic interplay of changes in all components of the face—bone, muscles, ligaments, deep and superficial fat compartments, and skin.^3,4^ In the midface, the suborbicularis oculi fat (SOOF), along with the malar fat (ie, medial, middle, and lateral temporal cheek fat compartments),^5^ provides structure and contour to the cheek.^6,7^ Age-related changes in the midface and periorbital areas contribute to the worsening of infraorbital hollows (IOHs).^8,9^ Patients treated for midfacial volume deficit with HA and other rejuvenation methods reported satisfaction with their overall facial appearance and in adjacent areas, like the TT.^10^ Increased skin laxity, and loss of structural (bony and ligamentous) support are also involved in the formation of the TT and palpebromalar groove (PMG).^1,11^

Hyaluronic acid (HA) dermal fillers are a popular option to improve the appearance of the under-eye area, and clinical studies have shown that they can efficiently attenuate infraorbital grooves and smooth the lower eyelid-cheek junction in selected patients.^12-18^ Based on mechanical parameters (ie, strength and stretch) and chemical components (ie, concentration, molecular weight, and crosslinker content), different commercially available dermal fillers containing HA show distinct viscoelastic and biophysical properties, which can be used to treat particular indications.^19,20^ Potential difficulties associated with HA filler treatment of IOH include: (1) thinness of the skin and the lack of subcutaneous fat in the TT, increasing the risk of implant visibility, discolouration, Tyndall effect (bluish discolouration due to light reflection and scattering in the gel's particles), or irregularities; (2) compromised lymphatic drainage exacerbated by inappropriate filler placement or use of highly hygroscopic materials; (3) repetitive contraction of the orbicularis oculi muscle (OOM) contributing to filler migration.^21,22^ Fillers intended for the infraorbital area should therefore be carefully selected and positioned to offer effective support but remain invisible at the skin surface and be placed in small quantities in the correct plane to minimize the risk of accumulation due to muscle movements.

In this narrative review, we discuss facial anatomy and age-related changes that contribute to the development of the TT and PMG and highlight key danger zones to prevent complications. We outline criteria for selecting candidates for IOH injection and describe direct and indirect treatment techniques and ideal product properties for achieving natural-looking results while minimizing complications.

## ETIOLOGY OF INFRAORBITAL HOLLOWS (IOHs)

Aging impacts all anatomical aspects of the periorbital region and results in several visible landmarks at the skin surface under the eye. It is important to clearly define the position of each of these landmarks to minimize procedural risks and avoid common pitfalls and complications associated with infraorbital filler injections.

### Definitions

**Figure 1.**
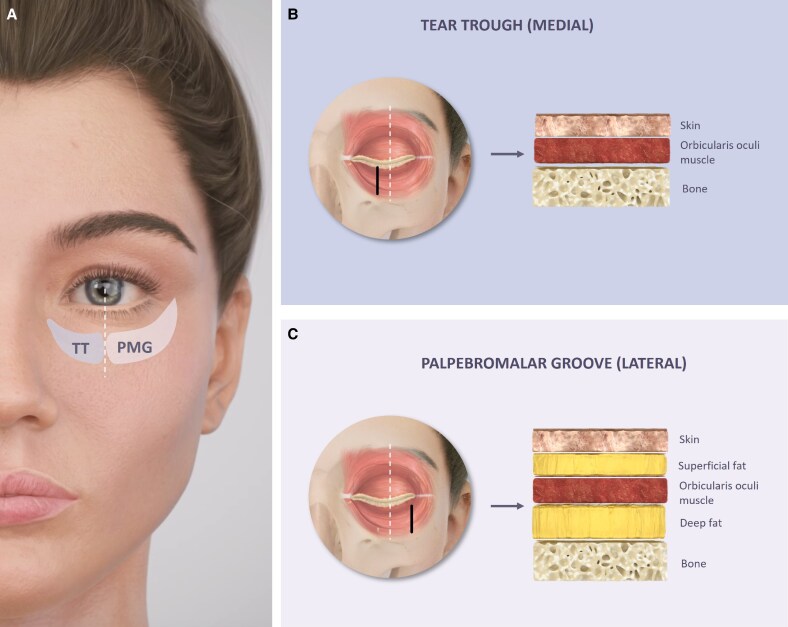
Infraorbital area. (A) Facial depiction of the TT and palpebromalar groove areas. Anatomical illustration of the TT (B) and the PMG (C), and their associated facial layers. PMG, palpebromalar groove; TT, tear trough.

The IOH are composed of the TT medially and PMG laterally (Figure 1).

The TT is a frequently overlooked major deformity of the orbital region, spanning a characteristic length of 2 cm, extending downward and outward from the inner canthus of the eye, giving a dissipated, unhealthy, fatigued, even haggard, appearance to the face, regardless of whether its boundaries are limited or extended.^23^

The PMG extends laterally from the mid-pupillary line and forms inferolaterally to the orbital rim. With aging, the TT and PMG may become a continuous groove, the *lid-cheek junction*, which demarcates the bulging orbital fat above the recessed mid-cheek below.^24^

The term *nasojugal groove* has been inconsistently used and often confused with the TT.^25-30^ The nasojugal groove is a surface depression, inferiorly to the TT, corresponding to the inferior border of the OOM.^31^ Therefore, the IOH may encompass a large area of depression occurring in the infraorbital area and at the lid-cheek junction.

### Anatomical Age-Related Changes by Layer

The face has 5 anatomical layers—skin, superficial fat, superficial musculoaponeurotic system, deep fat, and bone— although there are exceptions in the temple (10 layers), TT (3 layers), and preauricular region (7 layers).^32^ Surface anatomical landmarks such as the TT, PMG, and nasojugal groove may be visible even in young individuals or may become noticeable with age-related, multilayer changes.

### Bony Structure

The facial skeleton undergoes constant remodeling throughout life, thereby altering the location of the structures overlying the bone (ie, fat pads, ligaments, muscles).^33,34^ With aging, there is a pronounced maxillary bone resorption, recession of the inferior orbital rim, and overall widening of the orbital aperture.^35^ Resorption of the superomedial and inferolateral aspects of the orbit contributes to the signs of periorbital aging.^36^ Additionally, bony changes affect the orientation of the orbicularis retaining ligament (ORL) moving from a 90° orientation (young subject) to a 45° orientation (older subject) leading or contributing to the prominence of fat pads, deepening of the sulci, and evolution of eye bags.^1,37-39^

### Deep Fat

The SOOF is a layer of periorbital fat located deep to the lower lid OOM.^40^ This deep fat layer can be found bounded superiorly by the ORL and inferiorly by the zygomatico-cutaneous ligament (ZCL).^41^ The lower lid SOOF has medial and lateral components.^40^ The medial SOOF is situated deep to the OOM, extending from the mid-pupillary line to the lateral canthus, whereas the lateral SOOF runs from the lateral canthus to the temporal fat compartment.^42^ Computed tomographic assessments of the midface have shown that deep fat compartments atrophy with aging.^5^

### Muscle

The OOM is a widespread array of concentric muscle fibres encircling the upper and lower eyelids and situated beneath the skin, over the orbital septum, and the tarsoligamentous sling.^43^ It is anatomically divided into 2 main parts^1,11,44^: the orbital orbicularis, which controls voluntary eyelid closure and medial brow depression to protect the globe; and the palpebral orbicularis, further subdivided into the pretarsal orbicularis, responsible for eyelid closure during involuntary blinking, and the preseptal orbicularis, which manages eyelid closure during voluntary blinking and aids in tear drainage as part of the lacrimal pump.^1,45,46^ Both the pretarsal and preseptal portions of the OOM are almost devoid of superficial fat, whereas the orbital portion is covered by the superficial infraorbital fat compartment.^40^ The TT is anatomically positioned between the orbital and palpebral orbicularis, while the nasojugal groove corresponds to the inferior border of the OOM.^31^

With aging, the OOM undergoes several changes that contribute to the formation and exacerbation of the TT.^47-49^ The decreased tone of the OOM leads to reduced support for the overlying skin and fat, causing the skin to sag and making the TT more pronounced. Additionally, the muscle itself loses volume and undergoes atrophy, which exacerbates the hollow appearance in the infraorbital area. The weakening of the muscle affects the position of the orbital fat pads, leading to the descent or herniation of the infraorbital fat, contributing to the appearance of under-eye bags and deepening of the TT.

### Superficial Fat

Immediately deep to the dermis, the superficial fat compartments form a dynamic soft tissue layer that moves along with the underlying muscle.^36^ One of the essential superficial fat pads that contribute to facial aging is the *infraorbital fat* compartment. This structure is organized as 3 fat compartments (ie, inner, medial, and outer) found underneath the lower eyelid and beneath the orbital septum. With age, atrophy of the infraorbital fat and weakening of the septum, as well as the inferior and posterior drift of the inferior orbital rim, may contribute to fat herniation and worsening of the TT.^37,42^

### Ligamentous Structures

The infraorbital ligamentous structures include the TT ligament (TTL), the ORL, and the ZCL which all originate from the facial skeleton, pierce the muscle layer, and insert into the dermis.^50-52^ The tethering effects between their periosteal origin and their cutaneous insertion, cause the prominent grooves and troughs visible at the skin surface. The TTL and ORL with bony attachment of the OOM explains the TT and PMG, whilst the ZCL forms the inferior border of the zygomatic arch, giving the anatomical basis of eye bags malar bounds, and malar festoons.^38,50^

### Skin

The skin of the eyelid and infraorbital area is extremely thin, often <1 mm thick, with virtually no subcutaneous fat, making underlying structures such as blood vessels and muscles more visible.^44^ Additionally, the thicker cheek skin, supported by the medial cheek fat compartment, can accentuate the demarcation between the IOH and the cheek, worsening the indentation at the lid-cheek junction as aging progresses.^22^

Skin changes associated with aging, including reduced collagen and elastin production, result in diminished skin elasticity and firmness, leading to sagging and drooping, especially around the lower eyelids and cheeks.^53^

### Septa

The *malar septum* is a fascial network extending behind the OOM and originating from the orbital rim, at the same point that the orbital septum fuses.^54^ From its periosteal origin, the malar septum runs in a nearly perpendicular direction toward the skin while traversing multiple layers (ie, subcutaneous fat, muscle, and deep fat compartments). It inserts in the cheek, ∼3 cm below the external canthus thereby dividing the SOOF into an upper and a lower portion.^1,42^ The malar septum may create a poorly permeable barrier, so that fluids contained in this envelope do not spread into the cheek tissue.^55^ This is thought to be responsible for conditions such as persistent malar oedema and festoons.

The *orbital septum* is the thin sheet of fibrous connective tissue separating the intra- and extra-orbital structures.^56^ In young individuals, this septum lies perpendicularly, in a cranio-caudal orientation, emerging from the upper aspect of the orbital rim.^24^ Breaches of the orbital septum can result in prolapse of infraorbital fat and contribute to eye bags.

### Lymphatics

Lymphatic drainage of the under eye is dependent on the delicate lymphatic system that drains the skin of the lower eyelid to the preauricular and parotid lymph nodes. The infraorbital collecting vessels have been described as ravelling superficially (within the dermis and hypodermis), but a recent study uncovered another, deeper lymphatic network draining the conjunctiva and displaying connections with the former superficial network.^57^

With aging, lymphatic drainage efficiency diminishes, leading to increased fluid accumulation. Structural changes in lymphatic vessels contribute to decreased drainage capacity, while increased vessel permeability may cause fluid leakage and tissue swelling. These age-related alterations contribute to typical signs of aging in the infraorbital region, such as periorbital puffiness and under-eye bags.

### Danger Zones

**Figure 2.**
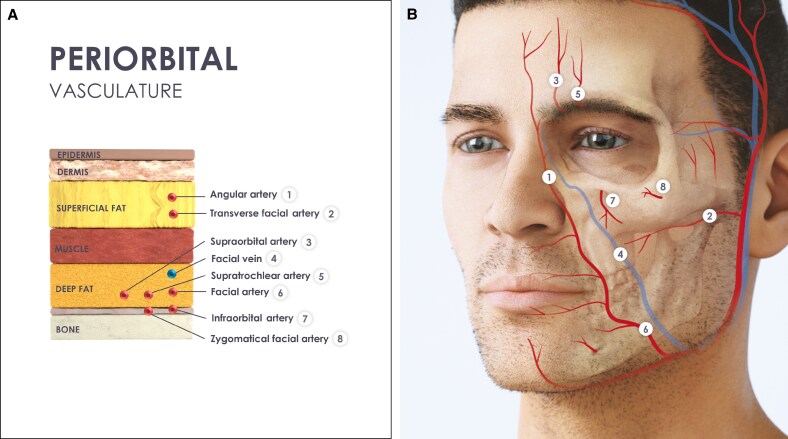
Vasculature of the periorbital area. (A) Cross-sectional illustration of the facial layers in the infraorbital hollow, showing the position of key blood vessels. (B) Illustration of the facial vasculature, highlighting key blood vessels to avoid during injections.

Understanding the course of the angular, infraorbital, and zygomaticofacial arteries is important to prevent intraarterial injection of foreign material that could have dramatic consequences, such as vascular obstruction, emboli, including transient or permanent vision loss in case of retrograde flow of the filler to the ophthalmic artery. For instance, a recent case study reported the first occurrence of orbital compartment syndrome following a filler injection in the TT, highlighting the potential for blinding complications.^58^ While this adverse event is rare, it highlights the importance of understanding anatomy and danger zones for safe injection techniques. Nonetheless, despite common beliefs, the infraorbital area is not associated with a higher risk of blindness compared with other areas of injection. Recent reviews have ranked the periorbital region a much lower risk than the glabella, nose, and frontal/temple areas in terms of most represented injection sites with reported blindness cases.^59,60^ The illustration of the periorbital vasculature is shown in Figure 2.

The *angular artery* (AA) is the terminal branch of the facial artery. After passing the upper part of the nasolabial fold, the AA goes up to the inner part of the orbit along with its matching vein. Periosteal injections performed in the TT and PMG theoretically are in general of very low risk of injury to this artery, caution should be exerted in the nasojugal groove area where the AA lies just above the OOM (ie, in the subdermal plane).^61-63^ The AA anastomoses with the dorsal nasal artery which is a terminal branch of the ophthalmic artery. Embolization along this vessel could thus compromise the blood flow to the nose and/or to the eye.^64^

The *infraorbital artery* (IOA) and nerve emerge through the infraorbital foramen located on the medial limbal line ∼0.6 to 2 cm below the orbital rim. The IOA divides into 2 to 3 main branches: the palpebral (observed in approximately one-third of subjects), nasal, and labial branches. The IOA commonly anastomoses with branches of the facial and ophthalmic arteries.^65^ The infraorbital foramen opens at a downward angle relative to the maxilla.^36^ Therefore, to minimize the risk of damaging the artery, this area should never be approached from below with a tilted angle.

The *zygomaticofacial artery* (ZFA) originates in the lateral SOOF, emerging from the zygomaticofacial foramen. The foramen is located ∼12 mm inferiorly and 9 mm laterally to the lateral canthus. Notably both the ZFA and foramen are absent in some subjects.^66-68^

The ZFA gives rise to many small branches (either at the foramen level or soon after) which can anastomose with the transverse facial artery. Together with the zygomaticotemporal arteries, they form a capillary network below the OOM. The capillary anastomosis is theoretically too small to constitute a potential HA embolus’ entry.^36^

## PATIENT ASSESSMENT AND SELECTION

A deep knowledge of IOH anatomy, combined with a comprehensive static and dynamic patient assessment during the physician–patient consultation, is crucial for tailoring the treatment plan and achieving the best possible outcomes. Three key metrics should be evaluated during the assessment phase: (1) the quantity of soft tissues in the midface and IOH; (2) the quality of the infraorbital skin; and (3) the severity of the hollow.^38,69^

### Quantity of Soft Tissue

Volume restoration in adjacent facial areas, such as the midface, may improve the appearance of the lid-cheek junction thereby reducing the volume of filler required to be injected directly in the infraorbital area.^9^ Evaluation should include examining the excess infraorbital fat above the lid-cheek junction, assessing the area below the lid-cheek junction, to identify midface volume deficits, specifically in the medial SOOF and deep medial cheek fat (DMCF), and loss of projection due to maxillary bone atrophy which may affect the lid-cheek junction’s appearance and contribute to dark circles.^69^ Excess lower eyelid skin can be a relative contraindication for filler injections, as the folds from the extra skin complicate accurate targeting and may limit the effectiveness of treatment. In cases with significant excess skin, patients might see minimal improvement even with substantial filler use.^38^

### Quality of Infraorbital Skin

Skin quality factors may exacerbate the appearance of IOH, such as skin thickness, laxity, hyperpigmentation, wrinkles, and oedema.^69^ Thinner skin can make dark circles more noticeable as it allows underlying pigmentation, dilated vessels, and the OOM to show through more easily.^70^ The aging process reduces skin elasticity and leads to excess skin, increased oedema, and sagging. These changes make dark circles more visible and wrinkles more pronounced, further emphasizing fat prolapse and deepening grooves.^25,71,72^

The quality and elasticity of the skin can be assessed using clinical methods such as the snap back test which measures lower eyelid laxity by timing how long it takes for the eyelid to return to its position after being pinched—a delay of over 2 to 3 s indicates severe laxity; the pressure test assesses the response of eyelid or surrounding tissues to applied pressure, helping to evaluate soft tissue volume loss vs skeletal support deficiency; the push test mimics a volumizing effect by displacing adjacent soft tissues, helping to anticipate the potential benefits of filler injections; and the lift test simulates a lifting effect by applying pressure on the zygoma, aiding in visualizing treatment outcomes.^38,73^

### Severity of INFRAORBITAL HOLLOWS

The severity, or size, of the IOH is important as very large, pronounced eye bags are more difficult to correct nonsurgically and are a relative contraindication for injection.^38^ According to the vector analysis, if the orbital rim is in front of the corneal apex, the vector is positive; if the orbital rim is behind the cornea, the vector is negative; and if it is in a vertical plane, the vector in neutral.^74,75^ Additionally, several descriptive grading systems, such as photonumeric scales,^76-78^ have been proposed to help the practitioner in the clinical evaluation of TTs and direct the treatment choice.^25,28-30^

The authors emphasize that it is also crucial to differentiate between various types of bags under the eyes. Bags of skin are typically due to skin laxity and sagging, while bags of fluid result from oedema or fluid retention. Bags of fat are caused by protrusion of the orbital fat pads, often due to aging or genetic factors. Bags from previous HA injections can occur if the filler migrates or is over-injected, leading to a swollen or uneven appearance.

### Candidates for Treatment

The ideal candidates are younger patients with mild to moderate eyebags, thick skin, and taut skin with minimal laxity. Patients with deeper grooves, fat prolapse, pronounced eye bags, thin skin, or significant skin laxity may not benefit from HA injections or may obtain only minor improvements. Some may have a higher risk of irregularity and filler visibility. Careful consideration should be taken of any pre-existing periocular oedema and addressed according to its etiology. Existing malar oedema is generally a contraindication for injections in the PMG. Patients with hyperpigmentation should be advised that HA cannot directly reduce the dark color of the overlying skin, but changing the concavity of the shadow can improve the darker appearance by reducing shadow.^1,25,38,79^

## TREATMENT PLANNING

When planning IOH rejuvenation treatments, a strategic approach is crucial. Correct assessment of injection depth and the appropriate layer for product placement is critical for successful infraorbital filling procedures, as incorrectly placed product may result in a number of immediate or delayed side effects.

A cadaveric study introduced the surface volume coefficient in facial fat compartments treatment effectiveness.^80^ The SOOF showed the highest efficiency—95% of injected product contributing to surface projection vs 26% for the DMCF. Additionally, the line of ligaments concept suggests lateral treatments primarily offer lifting, while medial treatments focus on volumization.^81,82^ Targeting lateral injections before medial ones reduces filler volume needed for symmetry, as lateral treatments reposition medially located soft tissues.^82^ Repositioning the medial midfacial soft tissues also repositions the lower eyelid-cheek junction by an average 0.49 mm.^83^

According to the authors, patients with significant midface volume loss may benefit from an indirect approach. Young patients can usually improve with deep fat compartment injections, while older patients often require and additional superficial treatment.^36^ It is recommended to first treat the midface deeply and laterally, then address the medial areas, and additional superficial treatment if needed. Direct treatment is suitable for isolated IOH without midface volume loss, while a combined approach may be necessary for pronounced TTs or persistent IOH after midface support.

When performing direct IOH treatment, filler placed superficially may create small “bumps” at the skin surface or a “Tyndall effect.”^84^ As mentioned above, when the filler is trapped between the malar septum and the skin, it may interfere with lymphatic drainage and encourage accumulation of fluid.^1,42,55,85^ Intramuscular injection of filler may also result in the product migrating, clumping, displacing, swelling and may increase the risks of nodule formation.^21^ Jitaree et al recommended that, to minimize the risk of arterial injury, injections should be performed into the supraperiosteal layer using an entry site located superolateral to specific landmarks (ie, at the inferior margin of the TT, located between the lateral limbus and lateral canthus; at or slightly lateral to the mid-pupil line; and at 0.5 cm of the TT from the medial canthus).^86^ Direct injection techniques should aim to place product as close to the periosteum of the orbital rim as possible to provide a long-term, natural-looking, and safe infraorbital rejuvenation.

Product volumes should be minimal (maximum 0.5 mL per eye) to reduce risks of oedema or surface irregularities and avoid “overfilling” effects, including swelling due to water attraction to the filler. In a systematic review including 23 prospective and retrospective studies, the average injection volume was 0.47 mL per side.^13^ Authors reporting on TT correction with fillers generally recommended minimizing injection volumes at the initial treatment mainly in consideration of the nature of HA to attract water.^38^ Under-correction followed by an optional touch-up injection several weeks after the initial treatment is the consensus.^1,87,88^ Periocular areas are prone to oedema even in the absence of HA. However, if the presence of HA is implicated in the cause of persistent oedema, the best action is to dissolve using hyaluronidase. The authors suggest that the removal of previously misplaced or migrated HA should be considered before administering new treatments. This ensures a clean and effective foundation for the new treatment.

There is currently no universally accepted protocol for reversing HA-based fillers, and the required hyaluronidase concentration depends on factors such as HA particle size, crosslinking degree and technology, and G′.^89^ The authors believe that, while ultrasound is a relatively new tool and highly user-dependent, it can enhance filler dissolution accuracy. However, HA filler can remain visible for extended periods, especially when encapsulated in muscle or trapped in the subcutaneous layer, and can often be clinically assessed without ultrasound.

## TECHNIQUES

### Indirect Treatment—Midface Lifting and Volumization

Indirect IOH treatment aims to provide structural support by injecting the HA filler into the deep fat layer to not only enhance the treated area but also to positively impact adjacent regions, such as the IOH.

The original Tri-Site Bolus technique involves injecting 3 boluses into the lateral SOOF (lSOOF), medial SOOF (mSOOF), and DMCF to create deep pillars supporting the superficial mid-cheek.^41,90^ The technique can be performed with a needle or cannula. With a needle, the injections are administered slowly at a 70° angle, adjusting the syringe position to flatten the bevel as much as possible to the bone, with volumes of 0.2 to 0.3 mL per fat pad.

**Figure 3.**
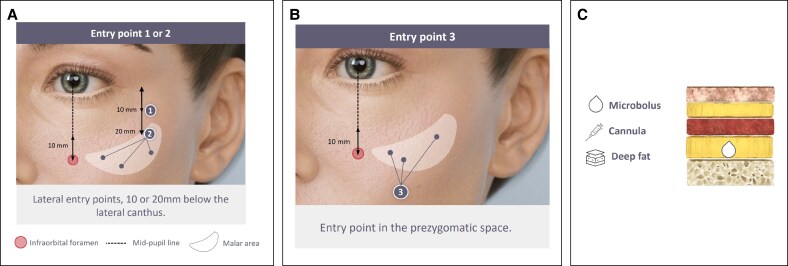
Indirect infraorbital hollow treatment (midface treatment to improve tear trough): multiple microboluses technique with a cannula. (A) Entry Points 1 and 2. (B) Entry Point 3. (C) Injection technique, device used, and targeted treatment layer, including a cross-sectional view of the precise layer of gel placement.

When using a cannula, several entry points can be used, including lateral and medial options (Figure 3A, B). The lateral entry point is located 1 to 2 cm below the lateral canthus, along a straight line from the lateral orbit to the lSOOF.^91^ The medial entry point is above the mid-cheek safety line, which runs from the medial canthus to the mandibular angle, in the prezygomatic space at the bottom of the DMCF.^62^ The cannula should be inserted at a 60° angle. Boluses are deposited in the mSOOF, lSOOF, and DMCF, for both injection devices.

Alternatively, the fanning technique involves horizontal injections with a cannula from similar entry points as the deep bolus technique.^36^ Using low pressure, filler is injected retrograde or anterograde in constant motion, delivering 0.1 to 0.3 mL along each track. The cannula is retracted and readvanced underneath the muscle, without complete withdrawal, targeting the different deep fat compartments. Care is taken to avoid advancing medial to the mid-cheek safety line to minimize risks of encountering facial vessels and overfilling the nasolabial fold region.

### Direct Treatment—Needle Techniques

**Figure 4.**
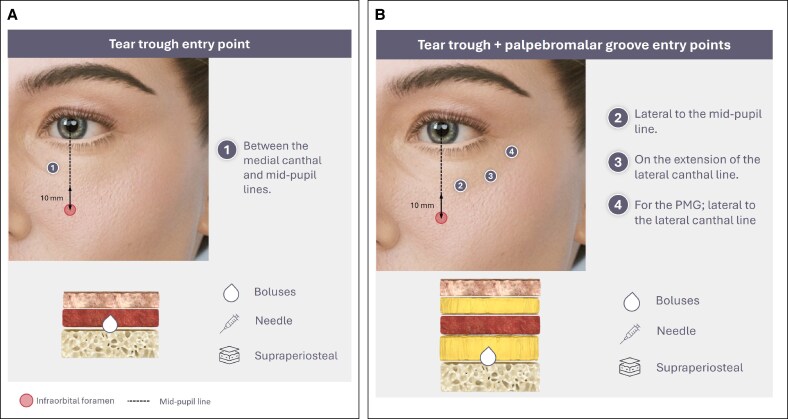
Direct infraorbital hollow treatment: serial punctures with a needle. (A) Entry Point 1 for tear trough (TT) treatment. (B) Entry Points 2, 3 and 4 for TT and palpebromalar groove treatment.

IOH may be efficiently restored using a serial puncture injection technique with a needle involving several small boluses evenly and closely spaced (Figure 4A, B).^92,93^ At the periosteal level, the area is free of significant vasculature. Concerns about the infraorbital foramen can be mitigated as it lies ∼1 cm under the orbital rim. Three different filling points on the orbital margin are recommended to improve the TT (Figure 4A, B): midway between the medial canthal line and the mid-pupillary line (1); slightly lateral to the mid-pupillary line (2), and on the lateral canthal line (3). If the PMG needs to be filled, a similar approach can be applied by choosing more lateral injection points (entry Point 4, Figure 4B).^91^ At each point, the needle should be inserted at a 45° or 90° angle while touching the periosteum; the bevel of the needle should be as flat as possible to the bone plane and then, a small aliquot (0.05) may be slowly deposited, at low pressure and bevel down and in contact with bone.

In addition, multiple micro-aliquots can be deposited at each injection point, by entering a few millimeters away from the area to be treated and directing the needle in the direction of treatment, and then retracting and redirecting delivering similarly sized droplets each time.^94^ If a bruise begins to form, the needle should be quickly withdrawn, and gentle pressure exerted with a cotton tip applicator or a gloved finger for at least 60 s to minimize subdermal bleeding.

**Figure 5.**
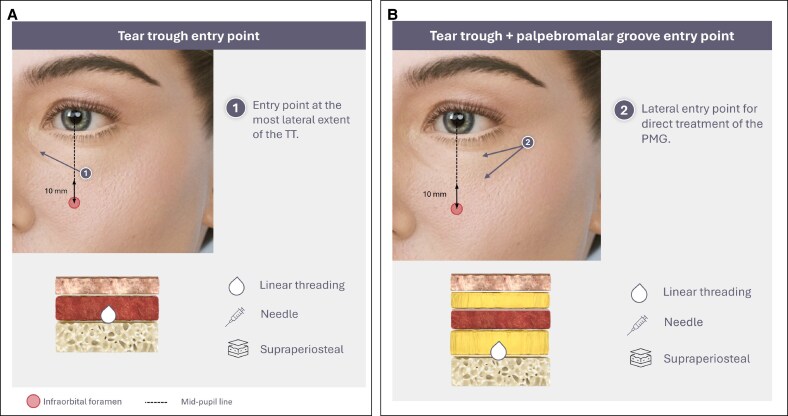
Direct infraorbital hollow treatment: linear threading with a needle. (A) Entry Point 1 for tear trough (TT) treatment. (B) Entry Point 2 for TT and palpebromalar groove treatment.

Linear threading with a needle is performed from an entry point at the most lateral extent of the TT, fully inserting the needle up to the medial aspect, following the inferior orbital margin, in a deep plane (entry Point 1, Figure 5A). The product can be placed in retrograde linear threads. Care must be taken not to exaggerate the orbital rim and enhance the hollow above. If required, the PMG can be addressed using a similar technique, accessing the area from a more lateral entry point (entry Point 2, Figure 5B).^23,95^

### Direct Treatment—Cannula Techniques

**Figure 6.**
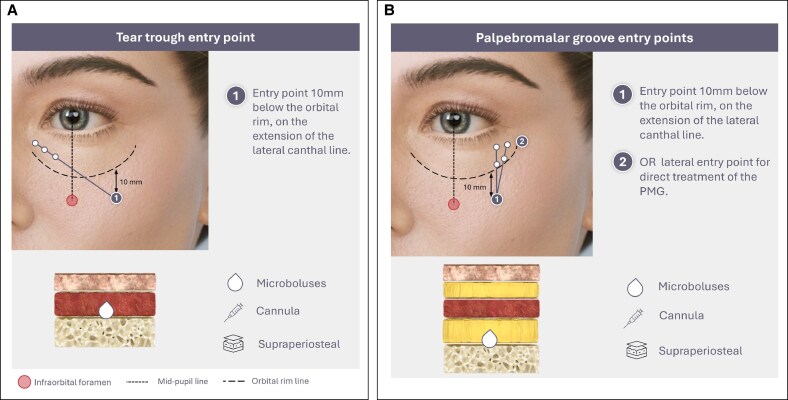
Direct infraorbital hollow treatment: microboluses with a cannula. (A) Entry Point 1 for tear trough treatment. (B) Entry Points 1 and 2 for palpebromalar groove treatment.

The IOH can be injected using a cannula in a retrograde or anterograde linear threading technique from an entry point in the SOOF, ∼20 mm below the lateral canthus, or 10 mm below the orbital rim (Figure 6A). This provides a safe and poorly vascularized area to inject both the TT and the PMG.^42,85,96^ The cannula is inserted through the entry point (its direction angled at ∼120° from the lateral to the medial aspect of the face) and glided within the deep submuscular/supraperiosteal plane until it reaches the inner point of the TT. The gel is slowly deposited as aliquots with low pressure using either a retrograde or an anterograde technique. The same or a second, more lateral entry point can be used with the cannula (Point 2, Figure 6A, B) to reach the PMG where the filler is injected deeply to create a lifting effect lateral to the lateral canthus under the OOM. Additionally, an entry point of 40 (approximately the length of a standard 25G cannula) or 50 mm from the medial canthus, aligned with the TT, is also viable.

**Figure 7.**
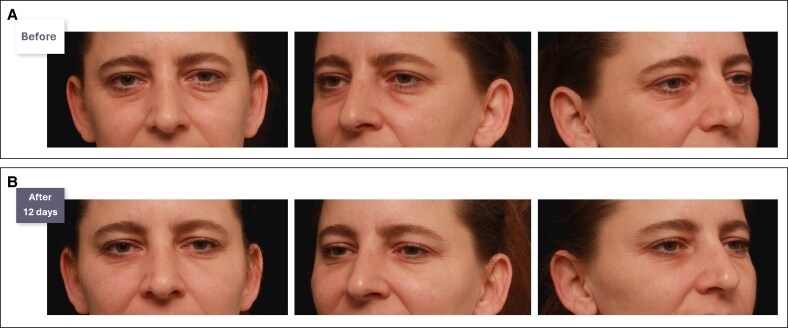
(A) Before and (B) 12 days after photographs of a 41-year-old female patient who received indirect and direct treatment with a hyaluronic acid (HA) filler to improve the infraorbital hollow. Treated indications included the zygomatic arch, zygomatic eminence, and malar area, with 0.2 mL per anatomical area; lateral midface (1.2 mL per side); tear trough (0.5 mL per side), and the lateral lid-cheek junction (0.35 mL per side). The patient exhibited hypertrophy of the tarsal portion of their periorbital muscle. Although HA filler treatment could be considered suboptimal, a clear aesthetic improvement is showed without the need for surgery.

**Figure 8.**
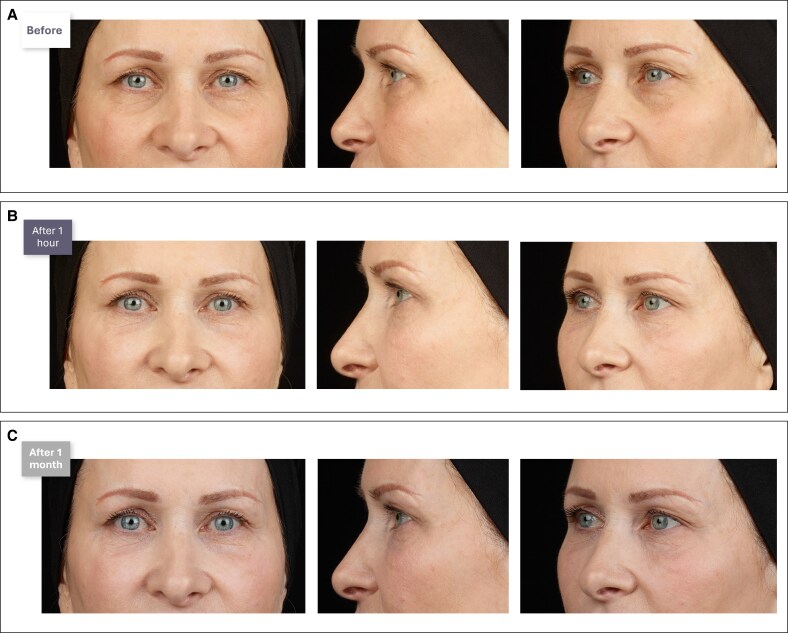
(A) Before, (B) immediately after, and (C) 1 month after photographs of a female patient who received indirect and direct treatment with a hyaluronic acid filler to improve the infraorbital hollow. A 56-year-old patient with moderate skin quality and mild photoaging, no skin hyperlaxity or lymphatic stasis, a negative vector and midcheek volume loss, presented for treatment. Treated indications included the malar area (right side: 0.5 mL; left side: 0.5 mL), and the tear trough and palpebromalar groove (0.5 mL per side).

**Figure 9.**
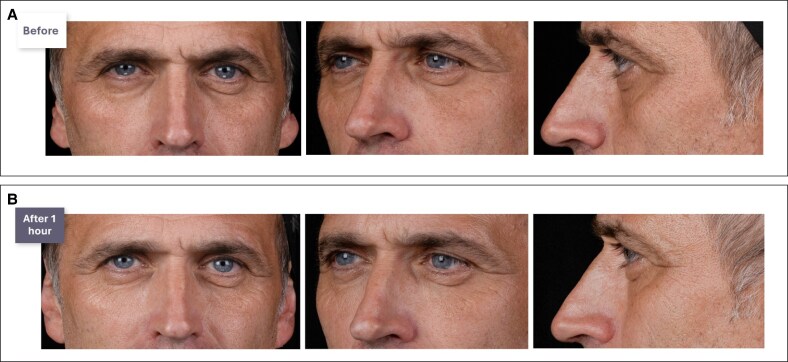
(A) Before and (B) immediately after photographs of a male patient who received indirect and direct treatment with a hyaluronic acid (HA) filler to improve the infraorbital hollow. A 41-year-old male patient with good, thin skin, and severe photoaging on the left side due to sunlight exposure presented for treatment. The patient had no skin hyperlaxity or lymphatic stasis but exhibited a negative vector and maxillary hypoplasia. The patient also showed midcheek volume loss. Treatment with HA dermal filler included the malar area (right side: 0.2 mL; left side: 0.7 mL), and the tear trough and palpebromalar groove (0.2 mL per side).

A step-by-step Video demonstrating the combination of indirect and direct IOH treatment is available. Clinical cases are presented in Figures 7-9.

## PRODUCTS

The anatomical complexities of the IOH require the use of a gel that is soft, easily mouldable, and resistant to accumulation over time due to overlying muscle contraction. It should be poorly hygroscopic to avoid attracting excessive water and minimizing the risks of post-injection oedema. The filler should require minimal injection force, even when using small gauge needles or cannulas, allowing for precise and slow deposits of small product volumes.^1,12,88,97^

To meet these criteria, fillers most suitable for IOHs should possess the following characteristics: low strength, (resistance to compression), and minimal swelling capacity (ie, high level of HA pre-existing hydration/saturation).^98^ They should remain spreadable and stretchable under the thin, soft tissue layers. This can be achieved by a combination of a low concentration (eg, 15 mg/mL) of a high molecular weight HA, with both non-crosslinked and crosslinked chains, with a limited degree of chemical modification to lessen the impact on the filler's fluidity and mouldability.^19,99^

## DISCUSSION

Despite the technical challenges associated with IOH filler treatments, the assumption that the IOH is an intricate area to treat is not supported by systematic reviews of reported filler complications. However, achieving a safe outcome using HA fillers in the IOH necessitates using specific techniques and informed product selection, as well as comprehensive patient assessment.

Effective treatment of the IOH should begin laterally with deep fat injection to achieve a lifting effect, progressing medially for volumization. This method not only enhances the overall midface appearance but also helps to attenuate the lid-cheek junction. Multiple boluses technique using a needle or a cannula, or a fanning technique using a cannula, can be implemented for deep fat treatment. If initial results are unsatisfactory, direct treatment of the TT and PMG can be performed using either a needle or cannula, targeting the supraperiosteal plane with techniques such as serial puncture or linear threading, depending on the injector's expertise. Entry point(s) may be adapted based on the treatment aim, always considering the infraorbital foramen's position.

The existing literature is consistent with our recommendations regarding patient selection and education, as well as expectable outcomes. A systematic review highlighted that supraperiosteal serial puncture and retrograde linear threading were the most common techniques described for infraorbital filler injection.^13^ The consensus was that fillers are usually placed supraperiosteally in the deep OOM, anterior to the inferior orbital rim. In a prospective trial involving 151 patients injected into the TT area, regardless of the device, investigators performed either serial puncture or retrograde linear injections, delivering an average volume of 0.48 mL per eye. Significant aesthetic improvements were reported, with only 27% requiring touch-ups after 1 month. Adverse reactions were mostly mild and transient, with rare occurrences of severe complications, resolved within 2 weeks without hyaluronidase.^100^ Diwan et al treated 25 patients using cannulas, delivering an average of 0.43 mL of filler per TT with a microdroplet and/or a linear threading technique. They reported that all patients noted an overall aesthetic improvement, with no major complications.^87^ Younger subjects with mild or moderate baseline defects reported higher satisfaction rates after treatment, which suggested that small volumes (limited to a maximum of 0.5 mL per eye in this study) may provide suboptimal improvement in older subjects with deeper grooves.^87^

In line with our recommendations, recent studies also indicate that injectors can choose between needles or cannulas based on personal preference.^101,102^ A randomized controlled study demonstrated that HA injections could be performed using either a depot technique with a 32 G 1/2-inch needle or a retrograde linear technique with a 27 G 1½-inch cannula.^101^ Similarly, Nikolis et al found that HA injections using either method result in similarly high efficacy and safety ratios.^102^ In another recent study showed that, at baseline, 73.2% of subjects received HA filler treatment with a 25 G cannula to correct IOH, while 26.8% were treated with a 27 G cannula. Thinner cannulas (eg, 27 G) are generally not recommended due to a false sense of security and higher risk of vascular accidents. The experts prefer injecting with 25 G cannulas for IOH treatments as they navigate tissue more safely and with less trauma.

While deep submuscular and supraperiosteal injections are widely recommended, Shah-Desai and Joganathan suggested that patients with thin eyelid skin might benefit from subdermal injections of a lighter, “skin-boosting” filler in the pre-septal segment of the TT.^1,13,38,79^ Promising results were obtained in young patients with dark circles who were treated with minimal filler amounts in a mesotherapy-like fashion. However, this technique may not effectively address infraorbital grooves in older patients, where volume loss is more global, and skin laxity precludes superficial filler placement. This approach may also increase the risks of bruising, oedema, Tyndall effect, and migration.

An important limitation of this work is that it consists of a narrative review, which lacks a systematic search, selection criteria, and quantitative analysis. This may introduce selection bias and limit the ability to comprehensively evaluate the evidence. Nevertheless, this review provides a valuable summary of considerations for the assessment and treatment of IOH to support injectors to achieve optimal outcomes.

## CONCLUSIONS

Safe, effective, and natural-looking rejuvenation of the infraorbital area with HA fillers is achievable in carefully selected patients. It involves a comprehensive approach that prioritizes midface lifting (laterally) and volumization (medially) before addressing the infraorbital area directly. The injector must have an in-depth knowledge of the regional anatomy, perform the treatment using the correct injection techniques, and select suitable products for this delicate area. The appropriate HA filler injected properly will safely and lightly fill the IOH while avoiding surface irregularities, swelling and discolouration.
